# Snus cessation patterns - a long-term follow-up of snus users in Sweden

**DOI:** 10.1186/s12954-020-00405-z

**Published:** 2020-09-10

**Authors:** Tove Sohlberg, Peter Wennberg

**Affiliations:** 1 Department of Sociology, Stockholm University, SE-106 91 Stockholm, Sweden; 2 Department of Public Health Sciences, Stockholm University, Stockholm, Sweden; 3 Department of Global Public Health, Karolinska Institutet, Stockholm, Sweden

**Keywords:** Snus, Snuff, Cessation, Patterns, Smoking cessation aid, Sweden

## Abstract

**Background:**

Several studies have investigated the role of snus as an aid to become smoke-free, but few have focused on who use snus, how they perceive snus use, why and how they quit, and their perception of being non-snus users. The purpose of this paper is to describe snus cessation patterns.

**Methods:**

Respondents are part of a 7-year follow-up of former smokers in Sweden. Initially, 1400 respondents were contacted regarding participation and 705 answered a web-based survey (response rate 50%). Out of them, 118 had used snus. The analyses include percentage distributions, as well as factor analyses of inventories, and configural frequency analysis in order to examine configurations of snus-related patterns.

**Results:**

Over 80% found snus of great importance to succeed with smoking cessation and half of them continued to use snus on a long term. Those who experienced both physical and psychological effects of switching to snus were the ones who continued and vice versa; those who did not experience such effects quit using snus. None made use of professional help but had their own strategies (60%), and most respondents who quit obtained psychological benefits (68%).

**Conclusions:**

The distinction between the concepts smoke-free, tobacco-free, and nicotine-free contributes to nuances in the debate on snus as harm reduction. Continued snus use does not mean that snus is not an effective aid to become smoke-free. Snus cessation is mostly mentioned in relation to advices on how to succeed, but the cessation process has rarely been described; therefore, this study expands the knowledge on this quite neglected topic and contributes to a more nuanced picture of snus cessation.

## Background

Swedish snus (moisturized tobacco meant to be placed under the upper lip) has been part of Sweden´s tobacco culture and commonly used for over a century. Since the mid 1980s, though, smoking prevalence has decreased and for some the use of snus plays a significant role in smoking cessation, while some go on to quit snus also.

Studies on prevalence, risks, and harm reduction as an argument for lifting the legal ban on snus in the European Union (EU) are relatively common. This study aims for a broader grasp of the process of becoming not only smoke-free but also snus-free, i.e., from initiation as an aid to giving up smoking to final cessation of snus as well.

### Snus - a Swedish speciality

Snus (sometimes referred to as “snuff”) is a moist tobacco either in loose form to pinch and place under the lip or in ready bagged portions (resembling tiny tea bags). This habit has been common in Sweden since the beginning of the twentieth century [1], but grew in popularity as an alternative to cigarettes when the negative health consequences of smoking began to attract attention. Today, 13.6% of all Swedes (22.6 and 4.6% women) use snus on a daily basis, and actually male snus use is more prevalent than male smoking [2]. Snus has a historically embedded place in the Swedish tobacco culture, and although the product has been banned within the European Union (EU) since 1992, Sweden has a permanent exemption from this ban.

Swedish smoking prevalence is internationally low (about 10%), but taking snus into the equation brings total daily tobacco consumption to 22% [2], more on a par with other countries’ tobacco consumption. In spite of this, Sweden has very low tobacco-related illness and mortality, a circumstance probably partly due to the widespread use of snus—sometimes referred to as the “Swedish experience”—and often used as an argument to promote snus as a means of harm reduction to current smokers [3].

### Snus as a smoking cessation aid

What may be described as self-managed smoking cessation is quite common [4–6], but as the epidemiological evidence of smoking-related harm has increased, so have various interventions emerged in the form of professional treatments or medicinal aids, e.g., the nicotine replacement patches and chewing gums (NRT). However, of the successful quitters in Sweden and Norway who actually made use of an aid, snus was the most preferred method [7, 8] especially among men [9]. But is snus a safe and effective cessation aid? NGOs like the Swedish organization Psychologists Against Tobacco [10] advise against using snus as a means of becoming smoke-free due to their perception that there is a lack of scientific evidence, which is a view shared by the European Commission [11] who claims that since there are no controlled scientific studies, snus as a smoking cessation aid is not an evidence-based method. Subsequent studies, however, have tested the efficacy of snus using randomized controlled trials and found that at early quit rates snus is superior to NRT [12] and that those using snus were more likely to quit smoking completely [13]. The quit rate for smokers is shown to be positively related to the use of snus [7, 8], and the chances of smoking cessation are in general higher for those who make use of this alternative tobacco product [14].

### Becoming a permanent user or a snus quitter

Using snus as a means to quit smoking may result in continued use [9, 15], but a switch to snus does reduce the health risks associated with cigarette smoking. One US study showed that modified risk information increased the likelihood of snus use among current smokers [16]. It is fortunate therefore that Norwegian general health practitioners are allowed to recommend snus as a smoking cessation aid [17]. Substituting smoked tobacco with smokeless tobacco, such as snus, can have positive health consequences even though it does not break the nicotine dependence. For persons desiring to quit nicotine dependence entirely, there are nicotine-free snus manufactured by both tobacco companies and, e.g., NRT producers. However, advice on how to become snus-free is largely limited to websites run by such actors as doctors online, pharmacies, and the quit smoking/snus line. Some of these have been reviewed by researchers or health care practitioners, but most are without references to any scientific studies. Moreover, studies on health effects of quitting after several years of daily use are quite rare (see however [18]).

In conclusion, several studies have investigated the role of snus in the smoking cessation process, but we know little about which users quit, why and how they do it, and how they perceive no longer being users.

### Aims

The aim of this study is to describe respondents who made use of snus as a smoking cessation aid and their perception of snus use, as well as why the snus quitters choose to quit, how they did it, and their experiences of being snus-free. A further aim is to determine factors influencing the decision of consumers to quit or continue using snus.

## Methods

### Data

**Fig. 1:**
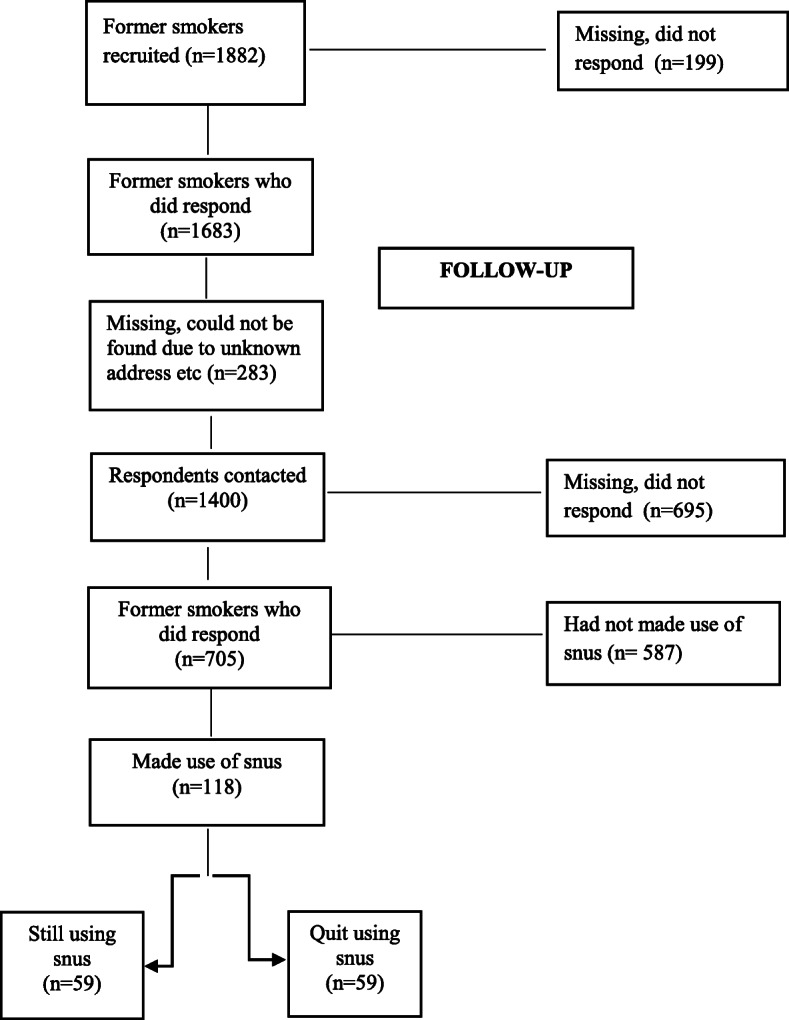
Flowchart showing respondent sample size

Respondents for this study were originally recruited from the Monitor project, a running survey conducted by the Centre for Social Research on Alcohol and Drugs (SoRAD, now within the Department of Public Health Sciences) at Stockholm University between the years 2000 and 2012. At the beginning of each month, 1500 respondents from a representative sample of the Swedish population aged 16–84 were interviewed by telephone—in total 18,000 per year. During the period October 2009–May 2010, a sample of former smokers was recruited for a cross-sectional study on smoking cessation (see Fig. 1). The response rate was over 89%. These respondents are now part of a 7-year follow-up with the overall aim to study factors associated with long-term smoking cessation on an individual level, e.g., the use of snus. Both studies were approved by the Regional Ethical Review Board in Stockholm (2009/2102-31/5; 2017/561-31/5). In all, 1400 respondents were contacted regarding participation and asked to answer a web-based survey that ran from August 2017 to February 2018. The response rate was about 50%. The initial sample frame consisted of the general Swedish population. Previous studies have shown that respondents that could only be reached by mobile phones do not differ substantially from the general population with regard to their alcohol consumption [19] or tobacco use [20]. A non-response analysis on the follow-up sample showed no significant differences between participants and non-participants in such sample characteristics as sex, age, and education.

All respondents included in the present study made use of snus as a means of becoming smoke-free and some were still using snus on a daily basis, while others had quit using snus. Percentage distributions and factor analysis were performed with SPSS, version 23, and configural frequency analysis with the statistical data analysis tool SLEIPNER, version 2.1. These analyses are described in more detail below.

### Descriptive analyses

In order to give a background description of the snus users and of snus use as such the respondents’ *sex*, *age*, and *education* are shown in Table 1. Respondents were asked to state their sex as being woman, man, or other. Only one respondent stated other and is left out of the analysis. Current age ranged from 37 to 87. Highest level of education was measured by a direct question, and the respondent could tick a box indicating secondary school, high school, or university. Moreover, *time since start of using snus*, *how long using snus* after smoking cessation, the perception of *necessity to use snus to become smoke-free*, *perceived risks* with daily use, the snus quitters’ personal *experience of using snus*, current snus users’ *degree of dependence*, as well as their *wish to quit* using snus are included.

**Table 1:** Sample characteristics on snus users background, snus use, and experiences of snus use. (%) *n* = 118

*All who made use of snus*	
**Sex** (*n* = 117)	
Men	77.1
Women	22.0
**Age** (present) median	65 (std11)
**Education**	
Secondary school (9 years)	22.9
High school	36.4
University	40.7
*Snus use*	
**Time since smoking cessation/start for snus use (years) median**	22.5
**How long using snus**	
1–4 weeks	1.8
1–6 months	3.4
6–12 months	6.8
More than a year	37.6
Still using	50.4
**Necessary to succeed with a smoking cessation**	82.3
**Perceived risks with daily use (no or small risk)**	
Mouth cancer	43.6
Heart and cardiovascular diseases	48.3
*Snus-free only (n = 59)*	
**Experiences of snus use**	
More positive than negative	46.6
*Active snus users only (n = 59)*	
**High degree of dependence (mean 7.9)**	87.3
**Wish to quit with snus**	
No	76.8

The time since start of using snus was calculated from the stated year of smoking cessation since they also stated using snus as a means to becoming smoke-free. The respondents were also asked how long they kept on using snus with the alternatives less than 1 week, 1 week–1 month, 1–6 months, 6–12 months, more than a year, and still using. Whether snus had been a necessary aid to becoming smoke-free was measured with a four-point scale where they could mark agreement with one of the following alternatives: absolutely or maybe (Yes) or hardly or not at all (No). In order to measure the risk perception of snus use, the respondents were asked to imagine the situation that they had been using snus daily for at least 10 years and then mark on a four-point scale whether they perceived the risk for mouth cancer or heart and cardiovascular diseases as great to no risk at all. No or small risk were then collapsed into one category. Experiencing snus use as positive or negative was measured with a five-point scale ranging from more positive than negative to more negative than positive. The categories more positive and slightly more positive than negative were then collapsed into one category.

Lastly, the means of *degree of dependence* for those still using snus and *wish to become snusfree* are shown. Nicotine is a dependence-producing drug, but the degree of dependence varies between individuals. We have used the Swedish quit smoking line (2013) measured of four questions: level of snus consumption, taking the first snus of the day within 30 min after waking up or after 30 min, type of snus (where the strongest is loose snus, thereafter portion then mini portion bags), and for how long the respondent can resist using snus (less than 2 h, more than 2 h, more than 1 day). The alternatives were numbered according to indications of dependence (from 1 to 4). Thereafter, they were summed to a scale from 4 to 12 where 4–6 indicates a quite low level of dependence and 7–12 a high level. The respondents were also asked whether they perceived their snus use to be a problem with the alternatives (Yes) they wished to become snus-free or (No) they were happy with the situation.

### Statistical analyses

In order to examine the reasons to quit using snus, the respondents were asked to state whether snus was used only for a period as a *smoking cessation aid*, and whether they quit due to *health problems* connected with their use of snus or for *other reasons*. Respondents were also asked to tick a box with different options indicating how they quit. *Professional help* included psychotropic drugs, information/support, psychological help, acupuncture, hypnosis, and the quit smoking line (which also offers help to snus users). *Own actions* included reviewing snus habits, setting a date for quitting, decreasing the use of snus, practicing not having snus under the lip, leaving the snus box at home, and quitting together with a friend or family member. *Other actions* included support from family/friends/colleagues, reading about others’ experiences on the internet, increasing physical training, and comfort eating of more food/sweets (Table 3). Respondents were also given the possibility to clarify these actions in text (not shown in table). Benefits and side effects of quitting the use of snus were measured with an inventory and are described in relation to the factor analyses below.

### Factor analyses

Two questions used inventories consisting of several items, where respondents could mark on a 4-grade scale whether they agreed with certain experiences and to what extent they had perceived certain benefits or consequences. These answers were factor-analyzed (principal components factor analysis; varimax rotation; eigenvalues > 1). There was a pre-understanding of a number of factors and the analysis confirmed this (see Appendix for domains, items, factors, and factor loadings).

Analysis of an inventory with 12 items concerning the respondents’ experiences of quitting smoking and starting to use snus generated three factors: *positive physical experiences*, *negative psychological experiences*, and *positive psychological experiences.* These factors are included in Table 2. Perceived experiences of snus cessation were assessed using an inventory of 11 items that also generated three factors: *psychological side effects*, *physical side effects*, and *psychological benefits*, included in Table 3.

**Table 2:** Prevalence of configurations among those using snus as a smoking cessation aid (*n* = 118)

Quit with snus	Physical positive experience	Psychological negative experience	Psychological positive experience	Observed frequency (*N*)	Expected frequency (*N*)	*p* (Bonferroni corrected)	Significant type/antitype
Yes	Yes	Yes	Yes	8	4.35	*ns*	
No	Yes	Yes	Yes	9	4.35	.031	Type
Yes	Yes	No	Yes	16	18.99	*ns*	
No	Yes	No	Yes	24	18.99	*ns*	
Yes	Yes	Yes	No	2	1.99	*ns*	
No	Yes	Yes	No	1	1.99	*ns*	
Yes	Yes	No	No	4	8.67	*ns*	
No	Yes	No	No	4	8.67	*ns*	
Yes	No	Yes	Yes	0	3.20	.039	Antitype
No	No	Yes	Yes	1	3.20	*ns*	
Yes	No	No	Yes	14	13.96	*ns*	
No	No	No	Yes	9	13.96	*ns*	
Yes	No	Yes	No	1	1.46	*ns*	
No	No	Yes	No	0	1.46	*ns*	
Yes	No	No	No	14	6.38	.004	Type
No	No	No	No	11	6.38	*ns*

**Table 3:** Reasons to quit with snus, ways to do it, and experiences of being snus-free (%)

Why (*n* = 59)	
Only smoking cessation aid	23.7
Experienced health problems	30.5
Other reasons	45.8
**How** (*n* = 42)	
Professional support	0
Own actions	60.0
Other actions^a^	24.0
**Side effects of not using snus**	
Psychological (*n* = 51)	9.8
Physical (*n* = 50)	30.0
**Benefits of not using snus** (*n* = 50)	
Positive psychological experiences	68.0

^a^Other actions clarified in text are not included in the table

### Configural frequency analysis

In order to find snus use-related patterns in the data, a configural frequency analysis (CFA) was conducted [21, 22]. Variables included were current snus use (yes/no) and the three factors—*positive physical experiences*, *negative psychological experiences*, and *positive psychological experiences*—dichotomized to yes/no. Every configuration was compared with expected values of a chance model using binomial test, and configurations that occurred significantly more often by chance were labelled “types” and configurations that occurred less often than expected by chance were labelled “antitypes”. *P* levels were adjusted using Bonferroni correction in order to reduce the risk of mass significance. The CFA was performed in the statistical data program SLEIPNER version 2.1 [23].

## Results

### Background

About 70% of the total sample actually managed to become smoke-free without any smoking cessation aid, but of the ones who made use of an aid, almost 17% used snus. Background data on the snus users and the respondents’ experiences of snus use are shown in Table 1.

As expected, more men than women made use of snus. The respondents’ median age at the time of the study was 65 years, and most had continued to study after compulsory school—indicating an older, quite well-educated sample. Since they quit smoking/started using snus over 20 years ago, they were about middle-aged, so the level of education supposedly refers to the point of starting using snus. Most of those who now are snus-free used it for more than a year and of the total a great majority in retrospect found snus of vital importance to becoming smoke-free.

Daily use was considered an eventual risk to health, but slightly less than half of the now snus-free respondents reported more positive than negative experiences of being a snus user. In this study, half of those making use of snus as an aid to becoming smoke-free were still using it on a daily basis, where about 76% stated a high level of dependence. However, they were overall happy with their situation and did not want to become snus-free.

### Who quits and who continues to use snus?

The variables’ current snus status, positive physical experience, as well as negative and positive psychological experience of quitting smoking and starting to use snus instead were included in a configural frequency analysis to explore profiles of snus users (Table 2). As an example to understand Table 2, a total of 8 individuals who had quit with snus had a physical positive experience and experienced both positive and negative psychological experiences. In a random model, only 4.35 individuals would have this configuration (expected frequency) compared to 8 individuals in the data (observed frequency). However, difference between expected and observed frequencies was not statistically significant when correcting for the risk of mass significance.

Two configurations were found to be significant “types”. First, current snus users reporting positive physical experiences as well as negative and positive psychological experiences of switching to snus were a more frequent type than expected by chance. Second, snus quitters who had neither positive physical experiences nor negative or positive psychological experiences were also a more frequent type than expected by chance. The combination of having quit using snus and having no positive physical experience, but having both negative and positive psychological experiences, were found as a significant “antitype”, i.e., less frequent than expected.

### Experiences of becoming snus-free

Of this group in the sample, half actually quit using snus. Why and how they did it and if it was worth it in terms of benefits and side effects is shown in Table 3.

About every fourth quitter stated that their snus use was only a means to becoming smoke-free. However, it was also quite common to quit using snus due to health problems such as high blood pressure, stomachache, or problems of the teeth and mouth. Most common, however, were other reasons, such as economy (too expensive) and that others (family, friends, colleagues) complained about the smell or felt the habit to be disgusting. Interestingly, some also stated in the open-ended answers that the problems involved in carrying snus to other countries and buying it abroad was reason enough to quit. No one stated that they had made use of any kind of professional support to help with qutting; they rather did it on their own or together with a friend or family member. A few started to eat more and/or increased their exercise routines; however, the open-ended answers revealed that a quite common way of becoming snus-free was simply to make up one’s mind and to quit without planning ahead. As snus-free, a majority (68%) experienced psychological benefits such as feelings of freedom, happiness, and being a good role model. Some also experienced psychological/physical side effects, such as irritation/depression/ restlessness, problems of concentration, insomnia, headaches and dizziness, and/or other such side effects such as craving for sweets, with resultant weight gain and/or upset stomach.

## Discussion

This study aimed to describe persons making use of snus in their smoking cessation process, and those who continued using snus, and more importantly, those who treated snus as merely a means to becoming smoke-free and thereafter quitting, why they quit and how they did it, including the experience of being snus-free.

Large differences between sexes in using snus as a means to becoming smoke-free were found, men being in the majority, reflecting current sex differences in the daily use of snus in Sweden. The definition of “snus as an aid” is somewhat dependent on for how long this aid is used in relation to smoking cessation. Most of those who now are snus-free used snus for more than a year, indicating a more permanent switch from cigarettes to snus. In retrospect, most found the use of snus of great importance for a successful smoking cessation. The overall increase in snus use has led to decreasing rates of cigarette smoking and related negative health consequences [24], and indeed, experts have found a 90% reduction in the mortality risks when using snus instead of smoking cigarettes [25]. This underlines the role of snus as a means to becoming smoke-free, even if the switch from cigarettes to snus sometimes means remaining a permanent user.

The use of CFA shifted focus from group level statistics to person-oriented analysis, exploring how snus status related to physical and psychological experiences of switching to snus when quitting smoking. It was predicted that if this experience was predominantly positive, the use of snus would be ongoing and vice versa and that predominantly negative experiences would lead to ceasing use of snus. As expected, current snus users had experienced both positive physical effects like improved fitness and looks and more energy, as well as positive psychological effects such as decreased craving for cigarettes. Some negative psychological effects were also reported, such as missing the smoking community or feelings of emptiness and deprivation. Some current snus users yearned for cigarettes but perceived that snus helped with that craving, and the switch also meant gaining better physical condition. Hence, switching to snus had a great impact on their lives, probably partly explaining the continued use. Those who quit using snus after some time did not report craving a return to smoking or they did not experience any positive effects of using snus so quitting seemed a logical step to take.

It is common to talk about the difficulties of giving up tobacco in more medical terms, such as nicotine dependence withdrawals. In this study, the snus users were classified with a high degree of nicotine dependence, but as shown, several physical and psychological factors also had an impact on whether to continue using snus or not.

About every fourth participant seemed determined to make use of snus only as an aid to smoking cessation, even though snus is not considered an effective aid by Swedish authorities due to a claimed lack of evidence [26]. Quite a few participants stated a reason to quit was some kind of health problem, but many gave other reasons such as too expensive, complaints from others, and the difficulties of carrying snus into other countries or of finding it to buy abroad—problems making snus use less amenable. In Sweden, while smoking is circumscribed by extensive anti-smoking laws and restrictions, being a snus user is easier. It is legal, can be bought in almost every store, and use is permitted in all official arenas.

Somewhat surprisingly, none of the snus quitters stated having made use of any kind of professional help. This could be due to lack of information, for while information on smoking cessation is widely spread via, e.g., health clinics, pharmacies, and actors such as NRT manufacturers; information on how to succeed with a snus cessation is limited and demands an active choice to seek such advice on various websites (see e.g. [27, 28]). Interestingly, though, their own actions tended largely to correspond with the advice found on these websites, indicating that this information is not experienced as “professional help”. Research on smoking cessation suggests that women tend to plan their cessation more often than men do [19] and since snus is a mainly male habit this could be one explanation for ease or not of cessation. As shown, those to whom snus of itself meant nothing quit and then experienced positive feelings of freedom and happiness. If the goal of smoking cessation was to become tobacco/nicotine-free and free from quite a harsh habit (in terms of costs to health and pocket and increasing stigma), then having taken a detour via snus, it is not so surprising that finally becoming free releases such feelings. The side effects experienced are in line with physical withdrawal symptoms, but could also be part of breaking a habit where you constantly put something in your mouth. To experience cravings and then replace the snus with sweets, for example, is quite a logical result, but some participants reported a consequence was weight gain.

### Limitations and strengths of the study

Results must be considered in light of certain limitations. First, while the Monitor data on which the present study is based constitutes a representative sample of the Swedish population, the market research company simply replaced those who could not be reached with others and the missing data was as large as 60% in 2010 [29]. The first study of former smokers (from 2010) consisted of 1683 respondents whereof 1400 were contacted for a follow-up, finally resulting in a sample of 705 (response rate around 50%). As the sample size is quite small, the risk of sampling errors is increased. Altogether, this raises issues of representativeness.

The sex bias mirrors the proportion of snus users by sex in the population, where far more males than females use snus. Further, the respondents’ mean age of 65 is relatively high, maybe reflecting the opinions of an older (male) generation that would perhaps be different in a younger sample.

Lastly, the time of snus initiation and cessation dates back in time but previous studies on smoking status have concluded that recall is usually accurate [30, 31]. This probably also is applicable to snus status.

Keeping this in mind, the data on snus use and cessation presented here are rather unique. Research on snus often focuses on eventual negative health effects and sometimes is presented as an argument for harm reduction. This survey has covered the snus cessation process from initiation to cessation and therefore has been able to offer some new insights into snus use as a means to quit smoking, and unbiased data and rationale on snus cessation.

## Conclusions

It is hard to discuss snus as an aid to becoming smoke-free without also raising the question of harm reduction. Of the former smokers who made use of snus, some quit while others continued, becoming smoke-free but not tobacco/nicotine-free. It is therefore of great importance to distinguish between the concepts smoke-free, tobacco-free, and nicotine-free. Provided that all who use snus as an aid have the desire to become smoke-free, some have set the goal of also becoming tobacco and nicotine-free, while others are quite satisfied with continuing using snus and remaining nicotine users. Since it has been shown that snus use, at the expense of cigarette smoking, decreases the mortality risk and other smoking-related ill-health, snus should be considered as a preferable product—even if resulting in long-term use. Continued (tobacco/nicotine) snus use does not necessarily mean that snus is not an effective aid to becoming smoke-free.

A large number of studies offer consensus on concrete advice offered on how to quit using snus, but the sources of this knowledge are somewhat unclear. This study shows that several factors interact in the decision to quit and that the ways to do so vary, indicating a need for more research on snus cessation in order to develop more nuanced methods and better support.

## Data Availability

The dataset used and analyzed during the current study are available from the corresponding author on reasonable request.

## Appendix

### Domain, factors, items and factor loadings

**Table:**

	Factor loading 1	Factor loading 2	Factor loading 3	Explained variance (%)
**Experiences when quitting with snus**				
*Psychological side effects*				37.2
Irritated, depressed, restless	0.657			
Insomnia	0.739			
Palpitations, dizziness	0.859			
Headache	0.882			
Concentration difficulties	0.829			
*Physical side effects*				14.4
Upset stomach/constipation		0.713		
Cravings for sweets		0.745		
Weight gain		0.790		
*Psychological benefits*				12.3
Happy and positive			0.571	
Felt like a good role model			0.423	
Felt a sense of freedom			0.792	
KMO = 0.741, Bartlett’s Test of Sphericity = 0.000

**Table:**

	Factor loading 1	Factor loading 2	Factor loading 3	Explained variance (%)
**Experiences of starting with snus**				
*Physical positive experiences*				36.3
Improved fitness	0.701			
Clearer complexion	0.499			
Improved teeth	0.386			
Felt fresh	0.756			
More taste and aroma	0.847			
More energy	0.822			
*Psychological negative experiences*				15.0
Missed the smoking community		0.763		
Missed not get a break during work		0.612		
Feeling om emptiness		0.826		
Missed the routine of smoking		0.831		
*Psychological positive experiences*				9.0
Appreciated not having to go outside			0.691	
Cravings for cigarettes decreased			0.847	
KMO = 0.794, Bartlett’s Test of Sphericity = 0.000				
